# Nuclear receptors: from molecular mechanisms to therapeutics

**DOI:** 10.1042/EBC20210020

**Published:** 2021-11-26

**Authors:** Daniel E. Frigo, Maria Bondesson, Cecilia Williams

**Affiliations:** 1 Department of Cancer Systems Imaging, The University of Texas MD Anderson Cancer Center, Houston, TX, U.S.A.; 2 Department of Genitourinary Medical Oncology, The University of Texas MD Anderson Cancer Center, Houston, TX, U.S.A.; 3 Center for Nuclear Receptors and Cell Signaling, University of Houston, Houston, TX, U.S.A.; 4 Department of Biology and Biochemistry, University of Houston, Houston, TX, U.S.A.; 5 The Houston Methodist Research Institute, Houston, TX, U.S.A.; 6 Department of Intelligent Systems Engineering, Indiana University, Bloomington, IN, U.S.A.; 7 Department of Protein Science, Science for Life Laboratory, KTH Royal Institute of Technology, Solna, Sweden; 8 Department of Biosciences and Nutrition, Karolinska Institutet, Huddinge, Sweden

**Keywords:** cancer, endocrinology, nuclear receptors, steroids, therapeutics

## Abstract

Nuclear receptors are classically defined as ligand-activated transcription factors that regulate key functions in reproduction, development, and physiology. Humans have 48 nuclear receptors, which when dysregulated are often linked to diseases. Because most nuclear receptors can be selectively activated or inactivated by small molecules, they are prominent therapeutic targets. The basic understanding of this family of transcription factors was accelerated in the 1980s upon the cloning of the first hormone receptors. During the next 20 years, a deep understanding of hormone signaling was achieved that has translated to numerous clinical applications, such as the development of standard-of-care endocrine therapies for hormonally driven breast and prostate cancers. A 2004 issue of this journal reviewed progress on elucidating the structures of nuclear receptors and their mechanisms of action. In the current issue, we focus on the broad application of new knowledge in this field for therapy across diverse disease states including cancer, cardiovascular disease, various inflammatory diseases, the aging brain, and COVID-19.

## Introduction

Hormones are signaling molecules produced in one part of the body that can travel great distances to regulate the functions of distant organs, tissues, and cells. While the existence of hormones had been established since the early 1900s, their mechanism of action remained unknown for decades. That changed in the late 1950s when Elwood Jensen began a series of studies to understand how estrogen regulated immature female reproductive organs [1]. At the time, the prevailing theory was that hormones, such as estrogen or one of its metabolites, functioned as enzyme cofactors in much the same way as many vitamins. To better understand how estrogens worked, Jensen made a technological breakthrough that allowed him to efficiently radiolabel estradiol and thus, track its fate when injected into animals in small, physiological amounts [2]. When injected into immature rats, Jensen found that, unlike most other tissues, female reproductive tissues known to respond to estrogens like the uterus and vagina somehow captured and retained the hormone. This was the first evidence of a hormone receptor [3]. Moreover, Jensen noted that the radioactive signal was mostly present in the nuclei of cells, suggesting the presence of a nuclear hormone receptor [3]. This seminal discovery was first presented in 1958 to an international congress in Vienna that consisted of a grand total of five audience members [4]. Jensen proceeded to demonstrate that the hormone estradiol was not further metabolized in these cells [5]. Work from David Toft and Jack Gorski using sucrose density gradients and proteolytic enzymes proved that the receptor was a protein [6]. The collective work of Jensen and Gorski went on to build the early molecular model of estrogen’s mechanism of action, whereby estrogens bound a protein that could exist in the cytoplasm and promote its shuttling to the nucleus where it bound DNA [7–9]. While long suspected, the estrogen receptor (ER) was not proven to be a transcriptional regulator until Bert O’Malley’s laboratory discovered that estrogen induced the synthesis of chicken ovalbumin mRNA and protein [10]. The concept that hormone-bound receptors could bind to discrete regions of the genome and regulate the expression of distinct subsets of genes was solidified by the combined work of Jensen, Gorski, and O’Malley as well as other pioneers in the field such as Jan-Åke Gustafsson, Keith Yamamoto, and Gordon Tompkins [11]. Their work during the enzymology era went on to demonstrate the existence of a family of ligand-inducible transcription factors that could regulate key aspects of reproduction and physiology. The ubiquitous expression of nuclear receptors as well as their pronounced biological effects drew large numbers of new scientists into this emerging field.

**Figure 1:**
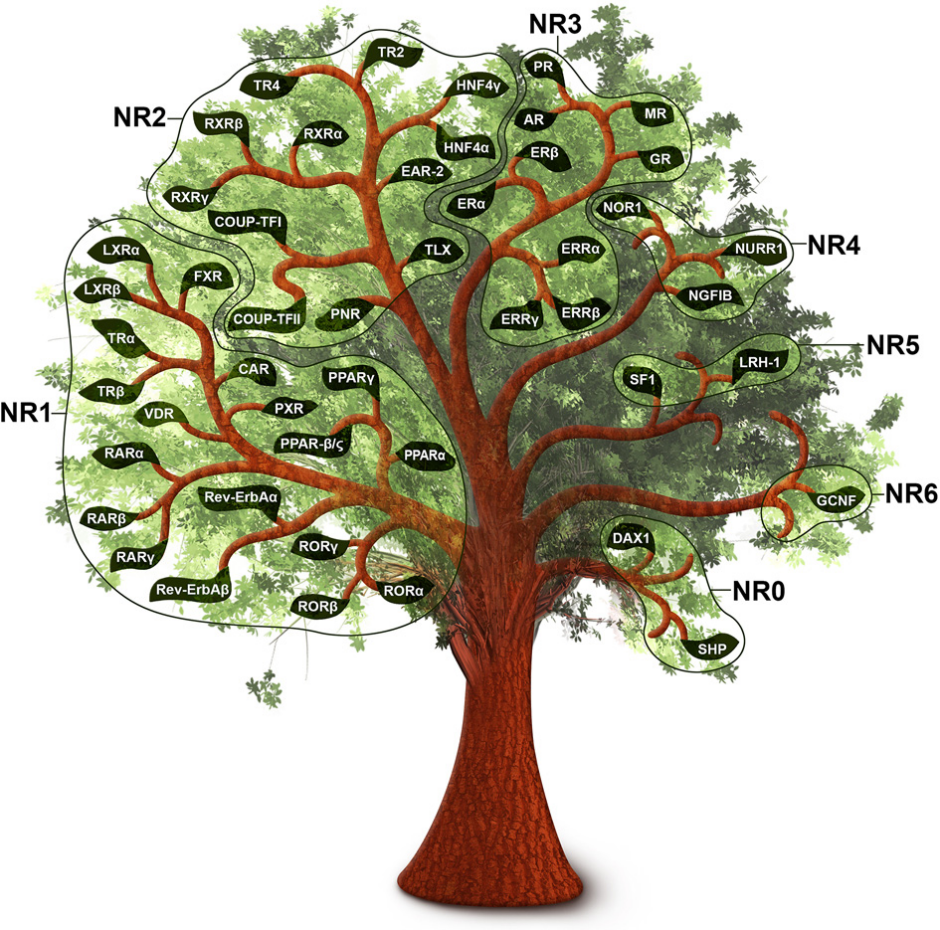
The nuclear receptor family tree All 48 human nuclear receptors clustered according to the Nuclear Receptors Nomenclature Committee-defined sequence homology subfamilies.

The advent of the molecular biology era in the 1980s led to a series of major breakthroughs for the field as the first nuclear receptors were cloned. The first full-length nuclear receptor to be cloned was the human glucocorticoid receptor (GR, encoded by the gene *NR3C1*) in 1985 by Ronald Evans's laboratory [12]. Around this time, Pierre Chambon’s group cloned the first estrogen receptor (ERα, encoded by *ESR1*) [13]. Both Evans et al. and Chambon et al. noted the similarity of these receptors to the viral oncogene *v-erbA* [14,15]. Also around this time, Evans and Björn Vennström, whose group had recently cloned the human complementary sequence of *v-erbA*, c-erbA [16], reported that c-erbA was a thyroid hormone receptor (TR, encoded by *THRA*) [17,18]. These findings merged steroid and thyroid hormone receptors into one family. A year later, cloning of the receptors for structurally diverse ligands including retinoic acid (a vitamin A metabolite) and vitamin D confirmed the existence of a large superfamily of structurally related receptors [19–21]. The formal defining of the Nuclear Receptor Superfamily was a major milestone for the field that launched a new era of endocrine physiology as it was now recognized that the development and physiology of diverse animal species were regulated by similar molecular mechanisms [22]. Following the sequencing of the human genome, we now know that there are 48 human nuclear receptors that, based on sequence analysis, are classified into seven subgroups (Figure 1). This large superfamily of proteins is thought to have evolved from a series of gene amplifications and subsequent mutations/diversifications. Most ligands for nuclear receptors are small, lipophilic compounds that can readily diffuse across the plasma membrane of cells and bind their cognate receptors, whereas other ligands, such as thyroid hormone, are actively imported into cells through specific transporters [23]. Although nuclear receptors were first identified as ligand-binding proteins, there is disagreement whether the common ancestor to all 48 receptors could in fact bind ligands or was a constitutively active receptor that bound DNA, possibly as a monomer [24–26]. It should not be entirely surprising then that more than half of the nuclear receptors do not have a known endogenous ligand. However, some of these ‘orphan’ receptors have been found to bind, often with low affinity, and be modulated by metabolites with previously unknown functions. Emerging evidence indicates that these ‘adopted’ receptors may have important functions in sensing metabolic changes [27].

The therapeutic potential of this field was realized relatively early on in the 1970s when Jensen and Craig Jordan demonstrated that an anti-estrogen called tamoxifen could be used to treat women with breast cancer in an ER-dependent manner [28]. These studies were later aided by the development of new antibodies that could detect the specific presence of the nuclear receptors. This work established one of the first examples of biomarker-driven, precision medicine [29]. Today, nuclear receptors remain one of the largest and most commonly targeted family of druggable proteins, accounting for billions of dollars in annual pharmaceutical sales [30]. Discussions of recent advances in the therapeutic targeting of nuclear receptors are presented throughout this special issue.

## Structure and mechanisms

**Figure 2:**
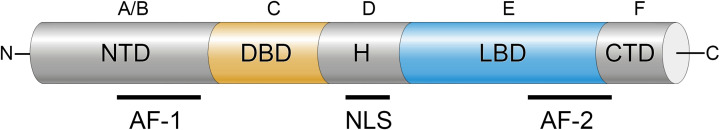
Nuclear receptor structural domains Nuclear receptors exhibit a common organization of their structural domains. This begins with the variable A/B domains that make up the NTD, which includes the first of two transactivation domains (AF-1). Next is the C domain, which contains a conserved DBD possessing two zinc finger binding motifs. C-terminal to this is the D domain, which is also known as the hinge region. The hinge region contains a short NLS. Next, is the fairly well-conserved carboxy/C-terminal E domain which contains the LBD as well as a second transactivation domain (AF-2). The E domain is particularly important for the dimerization of a group of nuclear receptors that form heterodimers. Finally, some nuclear receptors also possess a highly variable CTD (the F domain) that often has unknown functions.

A defining feature of nuclear receptors is their conserved functional domain organization (Figure 2). With the exception of the atypical nuclear receptors dosage-sensitive sex reversal-adrenal hypoplasia congenital critical region on the X chromosome, gene 1 (DAX1, encoded by *NR0B1*) and small heterodimer partner (SHP, encoded by *NR0B2*), all other nuclear receptors share four to five common domains labeled A–F. Domains A and B make up the highly variable (both in length and sequence) amino/N-terminal domain (NTD). This domain also contains the first of two transactivation domains (activation function 1, AF-1). The A/B domain is intrinsically disordered and hence, has made it difficult to obtain full-length, 3D structures of the receptors. However, as discussed here by Yi et al. [31] and Bourguet et al. [32], progress has been made in this area due to advances in crystallography and emerging technologies like cryogenic electron microscopy (cryo-EM) that can often more readily resolve large macromolecular structures. Adjacent to the A/B domain is the highly conserved C domain. This region contains the DNA-binding domain (DBD) which consists of two zinc finger DNA-binding motifs. The D domain or ‘hinge’ region is a flexible region that often contains the main nuclear localization sequence (NLS). C-terminal to the hinge region is the fairly well-conserved E domain which contains the ligand-binding domain (LBD). This region contains the second transactivation domain (AF-2). The E domain is often involved in dimerization. Finally, some receptors also contain a short, variable carboxy/C-terminal domain (CTD; F domain). While the F domain often has unknown functions, as described by Arao and Korach of this issue, recent studies indicate key roles for the F domain in ERα’s response to selective estrogen receptor modulators (SERMs) and more specifically, SERM-mediated LBD dimerization and AF-1 activity [33].

For many receptors, such as GR and the progesterone receptor (PR, encoded by *PGR*), in the absence of ligand, the receptors are held in the cytoplasm in an inactive state by heat shock proteins. Ligand-binding induces a conformational change that causes the release of the heat shock proteins, nuclear translocation, and dimerization (although the exact sequence of these two events, translocation and dimerization, is still debated and may be receptor- and/or context-dependent), and association with chromatin at specific sequences of DNA termed hormone response elements (HREs). For this class of proteins, often referred to as Type I nuclear receptors based on their mechanism of action, it has been realized in the past decade that the prevailing dogma that these receptors predominantly reside in the cytoplasm is often wrong. Instead, *in vivo* analyses indicate that in many cases, steroid hormone receptors such as ER and AR can almost exclusively reside in the nucleus, perhaps as a result of basal stimulatory signaling. Other receptors, such as TR are primarily found in the nucleus regardless of *in vitro* or *in vivo* conditions. Interestingly, recent studies have emerged that indicate rapid responses to some hormones such as progesterone and estrogen can occur via binding to and activation of extranuclear-localized receptors. In many cases, rapid signaling events can coordinate with more conventional genomic signaling to coordinate hormone-mediated gene expression [34]. For example, as described by Kerkvliet et al. of this issue, rapid stress signaling cascades can converge to phosphorylate and activate GR [35]. This increased GR activity, which can be ligand-independent, can promote positive feedback loops that maintain stress signaling and drive oncogenic cell behavior in triple-negative breast cancers.

Nuclear receptors exhibiting classic genomic effects can directly bind DNA as homodimers (e.g. GR), heterodimers (e.g. RAR/RXR), or monomers (e.g. SF-1, encoded by *NR5A1*). Additional DNA binding mechanisms such as tethering through other transcription factors is also common. As discussed by Arao and Korach, it has been revealed that some nuclear receptors such as ER, which classically bind as homodimers, can also bind DNA in a ligand-specific manner via long-spaced multiple direct repeat motifs or as monomers [33]. Studies from Gordon Hager’s group have revealed that nuclear receptors cycle dynamically on and off of DNA much more rapidly than previously thought [36]. Later, the omics revolution enabled large-scale studies of the impact of these receptors on the transcriptome, revealing how they bind over the genome to regulate their target genes. Studies from the early 2000s demonstrated that ER regulates hundreds to thousands of genes in breast cancer [37,38], and genome-wide binding-site studies pioneered by Myles Brown’s laboratory revealed that many ER enhancers were located far away from the genes they regulated [39]. Importantly, DNA response elements themselves, like ligands, can allosterically modulate receptor activity and secondary structure, as described for RAR/RXR heterodimers by Bourguet et al. [32]. Moreover, nuclear receptors are heavily regulated by post-translational modifications, providing yet another layer of transcriptional regulation.

Another breakthrough in our understanding of nuclear receptor mechanism of action was the discovery in the mid-1990s of coregulatory proteins that interacted with and modulated the transcriptional activity of nuclear receptors [40–46]. The discovery of these co-activators and co-repressors indicated that the previously held notion of nuclear receptors as simple, ligand-mediated on/off switches was outdated. Rather, nuclear receptors could function as inducible scaffolds capable of coordinating large transcriptional complexes. In that regard, different ligands could induce different receptor conformations which would lead to unique protein–protein interaction surfaces being exposed and therefore accessible for coregulator binding. This realization provides a framework for understanding how structurally distinct ligands such as SERMs could, via the same receptor, regulate different genes in the same cells. It also explains how the same ligand can regulate different genes in different tissues or developmental stages based on the tissue-specific availability of coregulators (reviewed in [47]). Receptor-bound coregulators can make direct interactions with the core RNA polymerase machinery and/or possess enzymatic activity that engenders the nuclear receptor–coregulator complex with the ability to modify chromatin structure and subsequent accessibility [48]. As described by Yi et al. in this issue, new insights from cryo-EM studies of ER and AR indicate that despite their similar domain organizations, these two related nuclear receptors interact with coregulators through different mechanisms—findings that will likely impact future drug development efforts [31]. A key feature of nuclear receptors is their ability to regulate different sets of genes within different cell types based on the tissue-specific transcriptional complexes they form and ability to select cell-specific enhancers. Formation of these cell type-specific complexes is often guided by lineage-determining factors that help open chromatin, allowing activated transcription factors to bind specific regions of the genome [49]. In this way, nuclear receptors can control patterns of gene expression that influence different aspects of physiology (e.g. reproduction and development) and disease (e.g. prostate cancer).

## Functions in physiology and pathology

Nuclear receptors are found in all multicellular organisms except fungi and plants. While the first nuclear receptors evolved approximately 640 million years ago in organisms that lacked organs or tissues (porifera), they likely functioned as environmental sensors. Endocrine or hormone receptors evolved later and are found in nematodes, insects, fish, and mammals [50]. They regulate key functions such as reproduction, development, and physiology. For example, the mineralocorticoid receptor (MR, encoded by *NR3C2*) is well known to modulate salt and water resorption in the kidneys through the ligand-mediated actions of aldosterone. MR also has additional important functions in adipose tissue, the brain, immune system, and heart [51]. In these tissues, MR can respond to diverse corticosteroids including aldosterone and cortisol. Here, aberrant MR activity is a causal driver of various pathological conditions such as heart failure. Accordingly, MR antagonists benefit patients with these conditions. Over the past decade, it has been recognized that there are important differences in MR regulation in the heart and kidney. As discussed by Young and Clyne in this issue, new regulatory mechanisms for MR that include both classical and non-classical rapid signaling have been described in the heart that have refined our understanding of how MR controls normal cardiac functions and can also contribute to heart disease [52]. Interestingly, the circadian clock has emerged as a novel partner of MR in the heart. These findings modify the current view of MR’s regulation and role in the heart and may have therapeutic implications.

While key functions for steroid hormone receptors like MR have been well established, our appreciation for the essential roles of orphan nuclear receptors such as Nur77 (encoded by *NR4A1*) has rapidly grown over the past decade. We now know that Nur77 is important for our adaptive and innate immune systems. In this issue, Lith and de Vries summarize the current knowledge on Nur77’s role in inflammation and highlight its potential in the therapeutic intervention of atherosclerosis, inflammatory bowel disease, multiple sclerosis, rheumatoid arthritis, and sepsis [53]. In addition, Safe et al. describe the therapeutic potential of Nur77, describing how novel, non-classical ligands bind different parts of Nur77, thereby enabling pharmacologic modulation of this orphan receptor [54].

The ERs (α and β) have critical roles in development (reviewed in [55]) and fertility. ERα controls neurobiological processes related to reproduction and ERβ is necessary for normal ovarian function and fertility [56,57]. ERα is also a mediator of secondary sex characteristics, including ductal elongation and growth of the mammary gland [58]. Since 1886, when it was first observed that oophorectomy (removal of the ovaries and thereby synthesis of estradiol) impaired metastatic breast cancer [59], the field has focused on modulating hormone signaling to combat this pathology. Following the isolation and characterization of sex hormones in the 1930s, when contraceptives were also developed, the synthetic estrogen diethylstilbestrol (DES) was shown to temporarily help some breast cancer patients [60]. This became the first true breast cancer drug. The mortality in breast cancer had been remarkably stable since the early 1930s (approx. 35 per 100 000 age-adjusted U.S. female population), but this changed during the mid-1990s and is now markedly reduced (approx. 20 per 100 000) [61]. This improvement coincided with the widespread approval and prescription of the SERM tamoxifen as a breast cancer treatment. Tamoxifen was synthesized by Dora Richardson at Imperial Chemical Industries, U.K., as a contraceptive pill in the 1960s, but failed [62]. However, inspired by the role of androgens in prostate cancer, further studies and clinical trials during the early 1970s showed its efficacy in the treatment of metastatic breast cancer [63]. While a patent in the U.S. was not awarded until 1986, tamoxifen is now one of the most prescribed therapeutics worldwide and is highly efficient both as an adjuvant and preventive therapy. In fact, tamoxifen was the first preventive therapeutic approved for any cancer [62]. Although its clinical development was not based on precise knowledge of its molecular mechanism, it is now known to function as an antagonist of ERα in the breast. Accordingly, the ERα gene (*ESR1*) was cloned from the human breast cancer cell line, MCF-7 [13,14,64], and studies by Jensen and Jordan in the 1970s demonstrated the therapeutic potential of targeting ER+ breast cancer with SERMs like tamoxifen [28]. Numerous ER-targeting drugs have since been developed and function by blocking estrogen synthesis, as ER antagonists (partial or full; for example, tamoxifen is an antagonist in the breast but agonist in the uterus and bone), or, more recently, targeting the receptor for degradation. ERα has correspondingly been used as a treatment-predictive biomarker in breast cancer clinics. Approx. 70% of primary breast cancers express ERα and are eligible for endocrine treatment. Even when as few as 1% of tumor cells express ERα, it is predictive of a response to treatment. Despite the success and broad arsenal of ER-targeting therapies available, resistance to these drugs remains a major clinical challenge. In this issue, Donald McDonnell and colleagues highlight novel mechanisms of resistance and potential strategies to overcome relapse by targeting reactivated ER [65]. Interestingly, the answer may not lie solely in targeting the cancer cell, but rather targeting ER’s non-cancer cell-autonomous roles.

Since the 2004 nuclear receptor issue of this journal, there have been tremendous advances in our understanding of AR's functions in prostate cancer, development of new AR-targeting therapies and identification of novel mechanisms of resistance to these hormone therapies. The major finding that came into focus around the period of the last issue was the realization that, despite the failure of current hormone treatments such as androgen deprivation therapy (ADT) and weak antiandrogens like bicalutamide (Casodex®), castration-resistant prostate cancers (CRPCs) still often rely on AR for disease progression [66]. As such, AR and the processes downstream of the receptor remain viable therapeutic targets. As a result, second-generation antiandrogens such as enzalutamide (Xtandi®), apalutamide (Erleada®), and darolutamide (Nubeqa®) have been developed and shown to delay disease progression and extend overall survival in men with advanced prostate cancer. Furthermore, the realization that adrenal androgens, as well as intratumoral androgen synthesis contributes to ADT resistance led to the development and now widespread use of improved CYP17A1 inhibitors like abiraterone (Zytiga®). These findings have changed the standard-of-care for patients at diverse stages of the disease. Beyond adrenal androgens and increased intratumoral androgen synthesis, several other mechanisms of resistance have since also been identified including *AR* gene amplifications, LBD point mutations, amplification of enhancers controlling *AR* gene expression, the presence of constitutively active AR splice variants that lack the LBD, and potential shifts away from AR dependence towards other cell lineages or even different nuclear receptors like GR. These newly identified mechanisms of resistance have reinvigorated interest in targeting nuclear receptors through novel approaches that are currently being tested in clinical trials. While these new discoveries have had a profound positive impact on men with prostate cancer, they have been extensively reviewed elsewhere and, as such, will not be covered in this issue. For more details on recent advances in prostate cancer, we refer readers to a recent book and two excellent reviews that more comprehensively cover this expanding field [67–69].

Since the revelations that both AR and ERα can be successfully targeted to treat cancer, the idea of pharmacologically targeting nuclear receptors for cancer treatment has been a major research focus. A second ER, ERβ, was discovered in 1996 by Jan-Åke Gustafsson's group [70]. ERβ binds the predominant endogenous estrogen (17β-estradiol, E2) with similar affinity as ERα. The two ERs have nearly identical DBDs. Still, they exhibit major functional differences, which are thought to be related to distinct differences in their AF domains and differential interaction with coregulators and other proteins. While major efforts have investigated the therapeutic potential of ERβ in breast cancer, this has been complicated by extensive use of antibodies with poor specificity [71,72]. Whereas ERβ is highly expressed in the ovaries (granulosa cells) where it impacts female fertility, it is now clear that ERβ is not highly expressed and thus does not appear to have a significant role in breast cancer. Conversely, other hormone receptors have been found to be therapeutic candidates for breast cancer, including GR [73], AR [74], and PR [75,76]. PR expression is regulated by ERα and has long been used as a biomarker for ER activity in breast cancer. However, studies have revealed a major role for PR itself. In this issue, Lanari et al. discuss current insights into the role of PR in mammary development and as a therapeutic target in breast cancer [77]. Moreover, female hormones impact human papilloma virus-driven cervical cancer. Sanghyuk Chung and colleagues describe recent mechanistic findings that elucidate how both ERα and PR impact cervical cancer tumorigenesis [78]. As noted by Haines et al., estrogen and its receptors also have anti-inflammatory properties [65]. Beyond estrogens’ actions in the breast cancer tumor microenvironment [65], Maioli et al. summarizes knowledge on how the ERs modulate neuroprotection and resilience of the aging brain [79], while Garcia-Villatoro and Allred describe ERs’ roles in colitis, inflammatory bowel disease, and colorectal cancer [80]. Finally, Ronald Evans's group portrays the view of bile acids as hormonally active compounds, synthesized from cholesterol in the liver and transformed by the microbiome, to act through nuclear receptors, notably the farnesoid X receptor (FXR, encoded by *NR1H4*), to regulate metabolic and immune homeostasis in the intestine [81]. He points to the potential to pharmacologically target this axis for prevention of metabolic disorders and intestinal tumorigenesis.

Because nuclear receptors evolved as, and still partly function as environmental sensors, they are targeted not only by endogenous ligands, but are also often unintentionally affected by other natural and synthetic compounds. This inadvertent targeting, known as endocrine disruption, is a growing concern because (1) dysregulation of nuclear receptor function and signaling often manifests as disease and (2) endocrine disrupting chemicals (EDCs) are becoming increasingly ubiquitous in the environment, impacting both humans and wildlife. Adverse consequences of EDC exposures include altered metabolism leading to diabetes and obesity, neurodevelopmental perturbations, reduced reproductive health, and increased risk of hormone-sensitive cancers in both women and men. Disturbingly, the increased disease risk can be inherited for several generations due to epigenetic modifications caused by altered nuclear receptor activity. Large-scale and high-throughput screening programs have been developed to perform the mammoth undertaking of identifying EDCs among tens of thousands of synthetic industrial and agricultural compounds [82]. Although endocrine disruption is not specifically discussed in this issue, it is important to note that EDCs are major contributors to nuclear receptor-mediated diseases. For a comprehensive review on endocrine disruption, see the Endocrine Society’s second scientific statement on EDCs [83].

This issue ends with a focus on the COVID-19 pandemic, which has ravaged the world during the composition of this issue, and its implications for nuclear receptor research [84]. The two research fields unexpected collided following early observations that (1) the expression of ACE2 and TMPRSS2, two host proteins that the SARS-CoV-2 coronavirus needs for infection, are regulated by multiple steroid hormone receptors and (2) clinical studies suggesting that antiandrogens may have a protective role in preventing the incidence and severity of COVID-19. This article reviews roles for steroid hormone receptors and how they impact COVID-19 in the contexts of lung development and function, the immune system, and expression of the host proteins, ACE2 and TMPRSS2. It concludes with a discussion of the potential for targeting steroid receptors to prevent infection or treat infected patients.

## Conclusions and future perspectives

Major strides have been made characterizing nuclear receptor mechanisms of action and their impact on biology. New technologies have enabled the 3D structure of full-length nuclear receptors in complex with coactivator complexes (cryo-EM) and comprehensive characterization of gene targets for individual nuclear receptors (omics). We now have a detailed understanding of nuclear receptor activation, their interactions with chromatin, transcriptional impact, and an appreciation of their non-genomic effects. Pharmaceutical interventions, such as those targeting ERα or AR, have improved cancer mortality for millions of people and, together with an array of other agents targeting a broad range of conditions such as hypothyroidism, inflammatory diseases, and metabolic syndrome, demonstrate the potential of nuclear receptor-based therapies. Work is ongoing to understand the biological function of each of the 48 receptors and how they impact diseases. This issue describes current advances and highlights the potential for novel treatments of multiple conditions.

Still, there are numerous poorly understood areas that warrant future investigations. These include studies that will permit a deeper understanding of the cell- and tissue-specific functions of nuclear receptors and the extent and mechanism of nuclear receptor cross-talk. Large-scale analyses and comparisons of nuclear receptors' cistromes and corresponding transcriptomes, proteomes, and metabolomes are prime for entering the new era of data-driven life sciences. Such efforts may help yield a systematic view and reveal the intricate manners in which nuclear receptors cooperate to regulate our physiology. In combination with new therapies for existing druggable receptors as well as manipulations of orphan receptors by novel ligands, we may continue the expansion of nuclear receptor-based personalized treatments. In future, we anticipate the continued design and development of superior therapies through selective targeting of multiple nuclear receptors that can simultaneously avoid adverse side effects—advances that will be enabled by a more complete understanding of how we can utilize cell- and tissue-selective treatments.

## Open Access

Open access for this article was enabled by the participation of University of Texas M.D. Anderson Cancer Centre in an all-inclusive *Read & Publish* pilot with Portland Press and the Biochemical Society under a transformative agreement with EBSCO.

## Abbreviations

ADT: androgen deprivation therapy
AF-1: activation function 1
cryo-EM: cryogenic electron microscopy
DBD: DNA-binding domain
EDC: endocrine-disrupting chemical
ER: estrogen receptor
GR: glucocorticoid receptor
LBD: ligand-binding domain
MR: mineralocorticoid receptor
PR: progesterone receptor
SERM: selective estrogen receptor modulator
TR: thyroid hormone receptor
